# Intraoperative Nerve Monitoring in Thyroid Surgery: A Comprehensive Review of Technical Principles, Anesthetic Considerations, and Clinical Applications

**DOI:** 10.3390/jcm14093259

**Published:** 2025-05-07

**Authors:** Ji-Yoon Jung

**Affiliations:** Department of Anaesthesiology and Pain Medicine, Konyang University Hospital, Konyang University Myunggok Medical Research Institute, Konyang University College of Medicine, Daejeon 35365, Republic of Korea; jiyooning1030@gmail.com; Tel.: +82-42-600-9317

**Keywords:** intraoperative nerve monitoring, thyroid surgery, neuromuscular blocker, recurrent laryngeal nerve, electromyography, anesthesia for thyroid surgery

## Abstract

**Background**: Intraoperative nerve monitoring (IONM) is increasingly recognized as an essential technique in thyroid surgery to preserve the integrity of the recurrent laryngeal nerve (RLN) and prevent postoperative complications. Although widely adopted, several technical and anesthetic factors can significantly affect the reliability and interpretation of electromyographic (EMG) signals. **Methods**: This narrative review summarizes the principles and methodologies of IONM in thyroid surgery, focusing on the mechanisms of RLN injury, the clinical benefits of IONM, and its limitations. Particular emphasis is placed on the anesthesiologic considerations, including the effects of neuromuscular blocking agents and anesthetic maintenance methods for EMG signal quality. Recent advances in alternative IONM techniques are also discussed. **Results**: IONM facilitates early detection of RLN injury and improves surgical outcomes. However, signal loss and technical errors occur in up to 23% of cases. Appropriate anesthetic management, such as the judicious use of neuromuscular blocking agents and the use of reversal agents like sugammadex, can significantly improve IONM accuracy. Alternative approaches, such as transcutaneous or thyroid cartilage electrode-based monitoring, show promise in overcoming current limitations. **Conclusions**: IONM is a valuable tool in modern thyroid surgery, aiding in the prevention of RLN injury. Anesthesiologists play a crucial role in optimizing IONM quality by managing factors that affect EMG signals. Ongoing research into novel monitoring techniques is expected to further enhance patient safety and surgical precision.

## 1. Introduction

Worldwide, the incidence rate of thyroid cancer after age standardization in 2020 was 10.1 per 100,000 in women and 3.1 per 100,000 in men. Additionally, the mortality rate was calculated to be 0.3 and 0.5 per 100,000 for men and women, respectively [1]. Since the early 1980s, the incidence of thyroid cancer has been increasing worldwide, but mortality is reported to be at a similar level or decreasing. These characteristics of epidemiology are interpreted to be due to the early detection of indolent cancer and the increase in overdiagnosis due to the development of screening tools [2].

Looking at statistics in Korea, thyroid cancer was the most frequent cancer from 2010 to 2014 in cancer registration statistics published by the Korea Central Cancer Registry (KCCR) based on a national and population-based database. But following widespread discussion about overdiagnosis, its reported incidence temporarily decreased, before rising again to become the most common cancer in 2019 [3]. In addition, thyroid cancer shows an excellent prognosis, with a 5-year relative survival rate of over 95.3% [4].

For thyroid cancer, the specific treatment method or treatment sequence varies depending on the grade, but in many cases, surgical treatment is recommended, and thyroidectomy is performed. In addition to malignancy, which is the most common cause, thyroidectomy can also be performed as a treatment method for benign disease, or hormonal disease that is not responsive to medical management [5]. Given the high incidence and favorable prognosis of thyroid cancer, preventing and minimizing surgical complications is crucial to improve long-term quality of life in patients.

Postoperative complications of thyroid surgery include major complications such as postoperative bleeding, hypothyroidism, recurrent laryngeal nerve damage and dysphonia, and wound-related minor complications such as seroma, scarring, and wound dehiscence [6].

Among them, recurrent laryngeal nerve (RLN) damage is reported to have an incidence of approximately 5–11% [7,8,9].

In most cases, damage to the RLN occurs unilaterally and is a temporary, non-life-threatening complication, with symptoms of voice changes and hoarseness [10]. However, these complications can also have a negative impact on patients’ quality of life, and since the RLN is related to the movement of the vocal cords, there is also a risk of vocal cord dysfunction, acute airway emergencies, and breathing difficulties [8,10]. In particular, bilateral RLN damage is rare, but if it does occur, vocal cord paralysis may occur, and permanent tracheostomy or gastrostomy may be required [11].

Therefore, preventing intraoperative RLN damage in thyroid surgery is an important issue. Intraoperative nerve monitoring (IONM) during thyroid surgery can be performed to identify and detect functional impairment of the RLN and vagus nerve during surgery. IONM monitors the electromyography (EMG) signal of the vocal cord muscles using surface electrodes of the EMG endotracheal tube. To minimize the occurrence of RLN injury through successful IONM, the influence of perioperative factors that may interfere with EMG signal interpretation must be minimized. In this review, the authors addressed the principles of IONM and the factors that can affect IONM signals for safe thyroid surgery.

## 2. Importance and Necessities of Intraoperative Nerve Monitoring

Intraoperative nerve monitoring (IONM) is a method of monitoring the anatomic and functional activity of the nerve of interest. By performing IONM, the distribution of RLN can be recognized more quickly than with the naked eye, and it also enables the surgeon to locate RLN easier and to predict nerve variation. Even if the RLN is intact when seen with the surgeon’s naked eye, invisible RLN injury may occur due to insults such as thermal, traction, and compression. IONM is helpful in detecting such injuries [12]. In addition, the dissection process can be less laborious, and functional integrity of RLN can be monitored.

There have been several studies that compared the incidence of temporary or permanent paralysis of the RLN between surgery with conventional RLN visualization and surgery with additional IONM [13,14]. According to a randomized clinical trial conducted M. Barczyński et al., transient and permanent RLN injury rates were significantly reduced when IONM was performed during the performance of a thyroidectomy [13]. Additionally, IONM enabled RLN localization before the nerves were visually identified and better detected bifurcation. It showed significantly favorable results in detecting bifurcation close to Berry’s ligament or inferior thyroid artery, demonstrating the effectiveness of IONM. However, another study has shown that IONM does not appear to affect the postoperative RLN palsy rate or predict RLN palsy [14]. However, results also showed that IONM appears to accurately predict good nerve function after thyroidectomy, evaluating IONM’s positive utility.

In addition to the findings that IONM has shown favorable effects in reducing RLN injury, another advantage is that it allows total thyroidectomy to be performed more completely. When performing total thyroidectomy, the process of dissecting the thyroid tissue around Berry’s ligament is surgically difficult, and RLN injury is likely to occur during this process [15]. When IONM is used during dissection and hemostasis, the use of instruments such as clamps or electrocautery—which may increase the risk of nerve injury—can often be avoided.

Recently, minimally invasive approaches to thyroidectomy have been increasingly adopted. These include minimal-incision open thyroidectomy, endoscopic, video-assisted, and robot-assisted techniques [16]. Compared to traditional open thyroid surgery, these approaches rely more on visual discrimination through the naked eye or monitor because it is difficult to palpate and magnify the tissue and nerves through surgical loupe due to the small incision [17]. In these cases, IONM can help determine the dissection plane and preserve RLN function.

## 3. Methodology of IONM

The International Neural Monitoring Study Group, comprising thyroid surgeons, anesthesiologists, electromyography (EMG) specialists, and laryngologists, presented standardized guidelines for the complex procedure of IONM [18].

These guidelines include the initial setting method of the IONM machine, the main causes and solutions when problems occur during IONM, the correct position of the endotracheal tube, and how to interpret the IONM signal. Their literature review suggested that preoperative laryngoscopy, presurgical dissection suprathreshold vagal nerve stimulation, postsurgical dissection suprathreshold vagal stimulation, and postoperative laryngoscopy are necessary for optimal IONM [18].

Various methodologies have been attempted for intraoperative nerve detection and monitoring, including placing postcricoid electrodes on the pharynx, use of an endotracheal tube with integrated electrodes, and monitoring of the cricopharyngeus muscle [19,20,21]. Among these, the current widely used method, owing to the advantages of safety, convenience, simplicity, and ease of standardization and consistent application, is the method using the surface electrode of the endotracheal tube [22].

**Figure 1:**
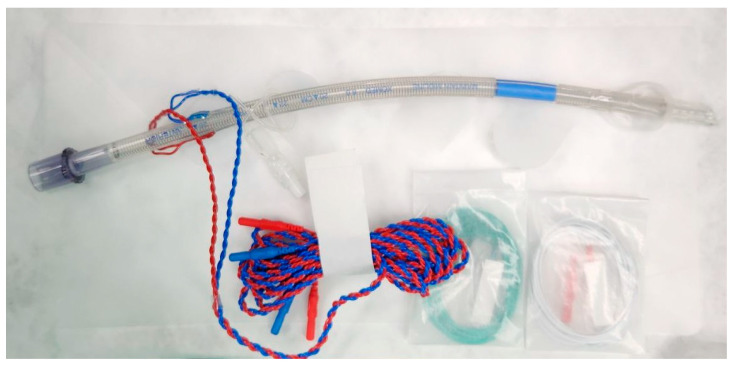
The specialized endotracheal tube for intraoperative nerve monitoring. The specialized Nerve Integrity Monitoring (NIM) endotracheal tube (Medtronic Xo med Inc., Jacksonville, FL, USA) incorporates imbedded bilateral stainless steel wire surface electrodes (Xomed ETT), assisting direct contact with the vocal cords.

The endotracheal tube is shaped as shown in Figure 1. A blue marked electrode is located on the proximal part of the balloon of the tube. If the colored band of this EMG tube is accurately positioned between the vocal cords, the function and integrity of the nerve can be monitored through the EMG signal during surgery [23,24]. Among the equipment that monitors acquired neural signals, the ones that are currently widely used include an audio-only system and a method that records both audio and visual waveforms. The latter can be effective in accurately quantifying and recording EMG for stimulation of the RLN and is also useful in discrimination between signals and artifacts [18]. Table 1 summarizes the standard procedures for IONM suggested by The International Neural Monitoring Study Group [8].

## 4. Problems That May Occur During IONM

With the assistance of IONM, RLN injury occurrence and postoperative complication incidence has reduced, improving the clinical outcome of patients after surgery. However, the incidence of IONM error is widely reported to be 3.8–23% [25,26]. According to the clinical guideline published by the Chinese Thyroid association [8], the types of IONM errors that can typically occur, and their solutions can be summarized as shown in Table 2.

If such errors occur during IONM, the surgeon runs the risk of making an incorrect decision based on wrong information obtained, the surgery time may be prolonged, and additional unnecessary maneuvers may be performed. Therefore, it is important to be aware of the types of errors that commonly occur and troubleshooting strategies so that the surgery can proceed smoothly. In particular, when there are problems other than technical problems, such as problems with anesthetic drugs and anesthesia methods, or problems with the placement of the endotracheal tube, the anesthesiologist must play an important role in solving the problem.

## 5. Anesthesiologic Considerations for Successful IONM

For optimal IONM, communication and cooperation between the surgeons and anesthesiologists are necessary. Although anesthetic management should be tailored to each patient’s demographic characteristics and underlying conditions, it must ensure adequate unconsciousness and analgesia while allowing reliable IONM signal acquisition. Additionally, an appropriate environment must be maintained in the surgical field so that the surgeon is not interrupted during surgery.

As the medications commonly used in anesthesia may interfere with adequate IONM, their selection should be guided by the type of waveform or potential to be monitored. Drugs used in almost all general anesthesia include inhalation agents, opioid, and neuromuscular blocking agents (NMBAs). Different monitoring modalities—such as electromyography (EMG) and motor evoked potentials (MEP)—vary in their sensitivity to anesthetic drugs. NMBAs significantly impair both EMG and MEP signals and should be avoided or reversed prior to stimulation. Inhalation agents, particularly volatile agents like sevoflurane, may depress evoked responses and are best minimized when MEP monitoring is needed. In contrast, opioids such as remifentanil do not interfere with EMG or MEP signals and are considered ideal adjuncts to maintain immobility and analgesia without compromising signal interpretation [18]. Table 3 summarizes the general effects of various anesthetic classes on IONM modalities.

### 5.1. Neuromuscular Blocking Agents

As mentioned earlier, the popular method of EMG detection and recording in thyroid surgery is recording the EMG signal detected in the vocal cord through an electrode attached to the endotracheal tube. Since IONM observes the EMG signal of the vocal cord muscles, loss of the EMG signal may occur when NMBA is administered in excessive doses during anesthesia [18]. NMBA is essential for full relaxation of the laryngeal adductor muscle and diaphragm to maintain good intubating conditions [27], and if intubation is performed without using NMBA, the risk of difficult tracheal intubation increases [28]. Therefore, it is advantageous to administer NMBA to prevent unnecessary muscle contraction during anesthesia induction and to obtain optimal conditions for tracheal intubation [29].

If the vocal cord muscles have not fully recovered from relaxation by NMBAs, the amplitude of EMG obtained by IONM becomes small and the sensitivity for detecting nerve damage becomes low [18].

The most widely used NMBA administered during induction of general anesthesia is rocuronium. This is a representative non-depolarizing neuromuscular blocking drug that allows rapid onset of muscle relaxation without the hyperkalemia, intraocular pressure elevation, and muscle pain that were problems with the depolarizing drugs used before [30]. The ED95 of vecuronium and pancuronium, another non-depolarizing neuromuscular blocking agent, is 0.056 mg/kg and 0.064 mg/kg, respectively [31]. In comparison, the ED95 of rocuronium is 0.3 mg/kg, which indicates a profile of rapid onset [32]. When rocuronium was given at 0.6 mg/kg, which is twice the dose of ED95, the drug onset in the adductor pollicis muscle was reported to be 60 to 90 s [33]. However, because the laryngeal adductor muscle is more resistant to NMB than the adductor pollicis muscle [34], optimal intubation conditions may require a longer time or a higher dose.

According to a previous study, the average time taken for the laryngeal adductor muscle to recover 90% after giving rocuronium 0.6 mg/kg, which is the dose required for tracheal intubation, was 34.9 min [27]. In another study on the pharmacokinetics of rocuronium, after injecting an initial bolus of 0.45 mg/kg, continuous infusion was performed to maintain T1 at 10%, and upon cessation, the times taken to reach T1 of 90% and TOF ratio of 0.7 were 31 min and 36 min, respectively [35].

In summary, although the neuromuscular blocking effect of rocuronium may interfere with accurate IONM by reducing EMG amplitude, its administration is necessary for intubating conditions. If the appropriateness of IONM is evaluated after sufficient recovery from the intubating dose of NMBA, and additional administration during surgery is avoided, NMBA will not be an obstacle to successful IONM.

However, changes in the pharmacodynamic characters of NMBAs may occur depending on the anesthetic agent used for induction and maintenance of general anesthesia. Previous studies have reported that the neuromuscular blocking effect can be potentiated depending on the use and type of inhalation agents [36,37,38,39,40]. These studies reported that the use of volatile anesthetics can augment neuromuscular blocking potency and have a moderate influence on the pharmacodynamic profile. Among various volatile anesthetics, desflurane and sevoflurane have the advantage of decreased solubility compared to their predecessors and are used in our center and most hospitals around the world. When these agents are used to maintain anesthesia, the effective dose of NMBA required for neuromuscular block comparable to that of total intravenous anesthesia or use of other agents was reduced and the duration of action was increased [36,38,39,40]. Also, although controversial, some studies reported that inhalation agents prolonged the recovery of NMBAs [36,40]. According to a study comparing the use of sevoflurane-based combined anesthesia and total intravenous anesthesia in thyroid surgery, the time to the first EMG signal for vagal stimulation after anesthesia induction was approximately 4 min faster in the latter group (median, 37.0 vs. 41.0 min, *p* = 0.028) [41]. Even in thyroid surgery where IONM is performed, nondepolarizing NMBA is used before intubation for safety and airway management, but additional administration during surgery should be minimized to recover normal muscle activity as quickly as possible. In these cases, compared to the appropriate use of NMBA during surgery, inhaled or intravenous anesthetic agents must be administered at a higher dose to suppress self-respiration, prevent bucking or movement without muscle relaxation, and prevent the surgical procedure from being interrupted. Even when a sufficient dose of anesthetic is used, propofol allows optimal IONM from the early stage of operation because the time until the first positive EMG signal appears is shorter than when using an inhalation anesthetic agent as mentioned above. Therefore, when performing IONM, total intravenous anesthesia can be more favorable than combined anesthesia as maintenance.

Another factor that can affect the action of NMBAs is old age. Elderly patients show increased AUC/dose and reduced total clearance of rocuronium, which has been reported to be associated with a decrease in creatinine clearance with age [42].

Patients taking other medications is another factor that may affect how NMBAs work. Antibiotics can cause depression of neuromuscular conduction because they have an inhibitory effect at the presynaptic and postsynaptic neuromuscular junctions [43]. Among them, aminoglycoside antibiotics show a greater effect of potentiating the action of nondepolarizing neuromuscular blocking agents by reducing acetylcholine release or blocking ion channels [44].

The antiarrhythmic agent that the patient is currently taking may also affect the action of NMBAs. When drugs such as procainamide, verapamil, and lidocaine are used for antiarrhythmic effects or to reduce cardiovascular changes during intubation and extubation, they can potentiate neuromuscular block [45]. It has been reported that this may be related to the mechanism of blocking the ion channels activated by acetylcholine and binding to the synapses of the neuromuscular junctions [46].

During general anesthesia, hypothermia can commonly occur due to physiologic changes in which heat loss increases, metabolic heat production decreases, and the compensatory response to these body temperature changes decreases [47]. In this hypothermic state, the duration of action and recovery time of NMBAs can be significantly prolonged, and the main mechanism is due to a decrease in elimination rate. In this hypothermic state, the duration of action and recovery time can be significantly extended due to a decrease in the elimination rate of NMBAs [48].

Therefore, in cases where NMBAs interfere with optimal IONM or affect IONM quality due to the above factors, reversal of NMBAs may be necessary.

Sugammadex is a gamma-cyclohexitrin-type drug that selectively binds to NMBAs such as rocuronium or vecuronium, preventing NMBAs from binding to receptors at the neuromuscular junctions [49]. Sugammadex can quickly reverse muscle relaxation and enable recovery of the TOF response within 2–3 min after injection, even in conditions of intense block [50,51].

According to the results of a randomized clinical trial, the time for electromyography of the laryngeal muscle to recover 100% after administration of 0.6 mg/kg of rocuronium was 26.07 min when 2 mg/kg of sugammadex was administered, and 50.0 min when waiting without administration of a reverse agent, showing significant difference. Additionally, when sugammadex was administered, it took 4 min for the train-of-four ratio to recover from 0 to over 0.9 [52]. Therefore, even if enough NMBAs are used for tracheal intubation, appropriate conditions for IONM can be created by promoting rapid recovery through sugammadex. However, the appropriate dose of sugammadex is still controversial. According to a randomized controlled trial, when comparing the cases of administering 1 mg/kg and 2 mg/kg of sugammadex immediately after intubation using 0.6 mg/kg of rocuronium, the former resulted in fewer unwanted bucking events while making the surgery field safer [53]. According to another study, for surgical relaxation and IONM quality, the administration of sugammadex at 0.5 mg/kg for deep block and 0.25 mg/kg for moderate block could increase the EMG amplitude of V1 stimulation [54]. In other words, the quality of IONM can be optimally maintained during thyroid surgery while minimizing unwanted involuntary movements by titrating the administration time and dose of sugammadex.

### 5.2. Placement of Endotracheal Tube

Electromyography endotracheal tube performs IONM with the function of mechanical ventilation during thyroid surgery under general anesthesia. As described previously, the exact location of the electrode attached to the surface of the EMG tube is very important because it monitors functions and integrities of the recurrent laryngeal nerve and vagus nerve. In other words, the EMG tube must be inserted at an appropriate depth and the surface electrodes of the vocal cords and the tube must be in optimal contact to exchange electromyographic signals and enable the analysis of the response of the vocal cord muscles.

Looking at the results of a prospective study on the causes of technical problems in IONM, the incidence of no EMG signal during initial vagal stimulation was about 10%, and most of these were cases where the tube was rotated or positioned too deeply [55]. In the same study, there were other causes such as too high insertion, improper tube size, and external wire disconnection, and in most cases, successful IONM was possible by adjusting the position of the tube to ensure optimal contact between the electrode and the endotracheal surface.

According to the 2022 American Society of Anesthesiologists Practice Guidelines for Management of the Difficult Airway, videolaryngoscopes are recommended for use as they improve laryngeal view and demonstrate higher frequencies of successful intubation and first attempt intubation, thereby confirming their efficacy [56]. In the population undergoing thyroid surgery, the presence of tracheal deviation or tracheal compression due to thyroid gland enlargement can present significant challenges for intubation [57]. Even in cases without a difficult airway, previous studies have reported that videolaryngoscopes provide a better glottic view, increase intubation success rates, and are associated with lower postoperative complications [58]. Due to these clear advantages, including safety and ease of use, the utilization of a videolaryngoscope for intubation is considered beneficial compared to the use of a direct laryngoscope.

It has been reported that for recording adequate electromyography signals using a tracheal tube-based surface electrode, it is crucial for the electrode to be correctly positioned at the level of the vocal folds. The use of a videolaryngoscope can be beneficial in achieving this accurate positioning [59]. In this randomized controlled study, the use of a videolaryngoscope significantly reduced the incidence of insufficient IONM signals and increased the tracheal intubation success rate compared to direct laryngoscopy.

During thyroid surgery, except for certain conditions where neck extension is contraindicated due to underlying medical conditions, a neck extension position is typically achieved by placing a roll under the shoulders to ensure adequate exposure of the surgical site. This positioning is also a critical factor to consider for the proper placement of the endotracheal tube. Previous studies have demonstrated that head and neck movement and alignment can influence the position of the endotracheal tube tip. In a study involving 20 patients, it was reported that during neck extension and flexion, the endotracheal tube tip moved an average of 19 mm away from and forward to the carina, respectively [60]. Another study reported that with 30 degrees of flexion and extension, the endotracheal tube tip moved 5.5 mm closer to or 6.3 mm further from the carina [61]. Therefore, even if the tube is positioned at an appropriate depth in the neutral position, changes in tube position due to the thyroid surgery position can result in the loss of IONM signals. It is essential to verify the correct positioning of the tube surface electrodes after positioning for surgery. If there is an EMG signal loss, adjusting the depth using a videolaryngoscope may be necessary.

One observational study compared the glottic views of videolaryngoscopy and direct laryngoscopy during tracheal intubation in the surgical position [62]. Intubation was performed in the thyroid surgical position with a specialized pillow placed under the head and neck. Regardless of whether external laryngeal manipulation was used to improve the glottic view, both the percentage of glottic opening (POGO) scale and modified Cormack–Lehane grade were improved with videolaryngoscopy compared to direct laryngoscopy. Based on this, it can be anticipated that using videolaryngoscopy in conjunction with thyroid surgical positioning prior to intubation may reduce the incidence of tube malposition or the need for repositioning. Although there is currently insufficient evidence to propose intubation after completing the surgical positioning as a standard protocol, further research is needed. Nonetheless, to ensure qualified IONM, it is essential to consider the potential migration of the tube due to positioning during airway management.

### 5.3. Avoidance of Signal-Interfering Substances

In addition to proper EMG tube positioning and anesthetic considerations, several practical techniques are essential for optimizing IONM signal quality. The use of lubricating gels or sprays containing local anesthetics—commonly applied during intubation—has been shown to interfere with electromyographic signal transmission from the vocal cords. These substances may impair electrode-to-tissue contact, reduce signal amplitude, or generate inconsistent waveforms, especially if applied directly to the recording surface of the endotracheal tube (ETT) [15,55,63].

Furthermore, the accumulation of secretions, particularly saliva or pharyngeal mucus, around the EMG electrodes can diminish the quality of the signal or lead to signal loss. To prevent this, routine suctioning and maintaining a dry surgical field are strongly recommended throughout the procedure [17].

Such technical precautions are included in several expert consensus statements and practical guidelines as part of signal optimization and troubleshooting protocols [18]. They contribute to maintaining consistent signal integrity and minimizing false-negative interpretations, which are crucial for safe and effective nerve monitoring.

## 6. Optimal Methods for Successful IONM

The ideal method for intraoperative neuromonitoring (IONM) should possess several key characteristics: strong EMG signals, non-invasiveness, stability, reproducibility, convenience, and a high positive predictive value. However, no current technique meets all these criteria perfectly. The EMG tube-based IONM method is widely used, despite being suboptimal.

The EMG tube offers advantages such as safety, convenience, simplicity, and ease of standardization and consistent application. However, its efficacy can be compromised by various factors, including the initial insertion direction and depth of the EMG tube, as well as changes in the contact area or degree of contact between the electrode and the vocal cords due to the patient’s head and neck movements.

In addition, the EMG tube is costly, and repetitive stimulation of the nerve during surgery is both burdensome and inefficient. Moreover, while the negative predictive value is high, the positive predictive value is relatively low and has been inconsistently reported, ranging broadly from 10% to 90% [64,65].

Therefore, to address these shortcomings and minimize intraoperative nerve injury, various alternative IONM methods have been developed and introduced.

To prevent loss of signal or errors caused by displacement of the electrode due to manipulation of the surgical site, one newly introduced method involves attaching adhesive skin electrodes to the skin at both upper margins of the thyroid cartilage for transcutaneous EMG recording. The feasibility and accuracy of this novel IONM technique have been studied in both porcine and patient models [66]. A prospective study on patients undergoing thyroidectomy compared the use of skin adhesive electrodes for IONM with the conventional EMG tube method. The results indicated that although the amplitudes obtained with skin adhesive electrodes were lower compared to the EMG tube, biphasic EMG signals with similar latency to those recorded by the EMG tube were achieved in scenarios with potential nerve damage [66]. This suggests that despite the primary limitation of reduced amplitude, skin adhesive electrodes could be considered a viable alternative to the EMG tube for IONM during thyroidectomy.

An alternative method recording EMG signals at thyroid cartilage electrodes has been reported as feasible, stable, and convenient in several clinical and animal studies. Anatomically, the thyroarytenoid muscles’ attachment to the inner surface of the thyroid cartilage provides a stable relationship that is not affected by surgical manipulation, making thyroid cartilage electrodes a viable alternative for assessing recurrent laryngeal nerve function.

In a comparative study, both EMG tube electrodes and thyroid cartilage electrodes were found to reliably record evoked laryngeal EMG signals [67]. The study showed that thyroid cartilage electrodes recorded significantly higher and more stable EMG amplitudes compared to EMG tube electrodes, with fewer false signals. This suggests that thyroid cartilage electrodes may offer a simple, inexpensive, and effective method for monitoring RLN function during thyroid surgery, presenting a stable and reliable alternative to EMG tube-based monitoring. While the use of thyroid cartilage electrodes offers the advantage of obtaining reliable and stable EMG amplitudes, it has limitations. One significant drawback is that these electrodes may obstruct the operative field. Additionally, this method may not be suitable for thyroid surgeries involving small incisions, where space is limited, and surgical access is constrained.

Despite the introduction of various tools designed to optimize intraoperative neuromonitoring (IONM), several challenges and limitations remain. Consequently, continued research and development are necessary to address these issues and enhance the efficacy and reliability of IONM techniques in thyroid surgery and other procedures.

## 7. Limitations and Practical Issues in Clinical IONM

While intraoperative nerve monitoring (IONM) offers significant benefits in thyroid surgery, particularly in minimizing the risk of recurrent laryngeal nerve injury, certain limitations remain, even when technical pitfalls are addressed. Issues such as EMG tube malposition, inadequate electrode–vocal cord contact, and signal loss or inconsistency have been widely recognized and are increasingly well-managed in clinical practice [18]. However, beyond these correctable sources of signal loss, broader limitations continue to affect the consistency and utility of IONM.

Anatomical variability presents a significant challenge. Variations in the course and branching pattern of the recurrent laryngeal nerve, as well as individual differences in laryngeal anatomy, may affect the positioning of electrodes and contribute to signal inconsistency or misinterpretation [68].

Also, cost and availability remain barriers to widespread implementation, particularly in low-resource settings. The expense associated with disposable EMG tubes, specialized equipment, and training may limit the accessibility of IONM, despite its potential benefits in reducing nerve injury and reoperation rates [69].

As such, successful IONM depends not only on technology but also on meticulous technique, interdisciplinary communication, and familiarity with both the strengths and limitations of the monitoring system.

## 8. Conclusions

Intraoperative nerve monitoring (IONM) is now widely used in thyroid surgery to reduce the risk of recurrent laryngeal nerve injury. While the technique has clear benefits, its effectiveness depends on multiple factors, especially how anesthesia is managed during the operation. Neuromuscular blocking agents, the depth and position of the endotracheal tube, and the choice of anesthetic maintenance method can all affect the quality of EMG signals.

Currently, EMG tube-based monitoring is the most common approach, but it has limitations—technical errors and signal loss still occur. In some cases, newer alternatives like skin electrodes or cartilage-based recordings may provide better signal stability, though more evidence is needed before they can replace standard methods.

Looking forward, refining anesthetic techniques and continuing to develop alternative monitoring technologies will be essential. Future research should focus not only on improving signal reliability, but also on making IONM more accessible and consistent across different surgical environments. Collaboration between surgeons and anesthesiologists will remain a key part of that progress.

## Figures and Tables

**Table 1:** The standard procedures for IONM suggested by The International Neural Monitoring Study Group.

Procedures	Detailed Procedures	Note
**Preoperative fiberoptic laryngoscopy**		
**Equipment setting**	The ground electrodes under the skin at the shoulders or xiphoid	
	Check electrode impedance and differences in impedance values	Electrode impedance < 5 kΩ, with deviations < 1 kω
	Check initial EMG	Initial fluctuations: about 10 μV
	Set up event thresholds	Typically, 100 μV
	The current intensity of stimulator probe should be routinely set at 1–3 mA	
**The monitoring device should be placed far away from electro-surgical devices and connected with anti-jamming silence detectors**		
**Confirm the recording electrode positions during surgery**	At the antemedial laryngeal line using stimulator probe	
**Four-step IONM**	Step 1: V1 signal	Obvious bipolar EMG signal is obtained at the ipsilateral vagus nerve at the plexas thyreoidea inferior level or at the plexas thyroid superior level in case of the presence of non-recurrent laryngeal nerve.
	Step 2: R1 signal	Before the exposure of RLN, its EMG signal is located by applying the probe vertical and parallel to trachea
	Step 3: R2 signal	Continuous monitoring is applied during the dissection of RLN in a real-time manner. After exposure, the most proximal end is detected for EMG signal
	Step 4: V2 signal	After complete hemostasis, the EMG signal of the vagus nerve is detected before closing the incision
**Signal analysis**	Basic EMG parameters	The biphasic waveform should be differentiated from the monophasic artifacts
	No obvious decrease in R2 and V2 signals	The basic EMG parameters include amplitude, latency and duration; the RLN has intact function
	Loss of R2 and V2 signals	If the RLN is injured, detect the “injury site” and the injury cause
**Photo recording the exposed RLN during surgery**		
**Postoperative laryngoscopy**		

EMG—electromyography; IONM—intraoperative nerve monitoring; RLN—recurrent laryngeal nerve.

**Table 2:** The types of typical IONM errors and their solutions.

Common Errors	Causes	Solutions
Too high electrode impedance	Technical defects in the electrode itself	Replace the electrodes
	Connection problem between electrode, interface-connector box, and monitor	Check the connections among equipment
	Contact problems of the subcutaneous electrodes	Check whether the subcutaneous electrodes fall off
Single electrode impedance > 5 kΩ	The surface electrode of endotracheal tube displaced	Intubate using videolaryngoscope
Impedance deviation > 1 kΩ	Insulating lubricant applied to the endotracheal tube	Avoid the application at the recording electrodes
Zero electrode impedance	Contact of two subcutaneous electrodes	Adjust to maintain a gap of at least 1 cm between the two electrodes.
Electrosurgical interference	The anti-jamming probe not connected	Circle the cable of the device, with the anti-jamming detector clipped on the twisted cable
V1 signal is absent	The vagus nerve injury	Directly detect the carotid sheath at 3 mA to obtain the V1 signal
	Present of non-recurrent laryngeal nerve	Recheck at the plexus thyroid superior level
	Improper anesthesia induction /muscle relaxant use	Wail until the muscle relaxant wears off or use an antagonist
	The detection current is not high enough	Check the connections among equipment
	Too low frequency of stimulus pulse	Stimulus pulse frequency: four times/s by default
	Too high event threshold	Routinely 100 μV
	Improper monitoring mode, channel, and volume	Recheck the setup
	Too short duration of detection for nerve	Each detection should be maintained at least 1 s
	Damaged probe, with insulation layer falling off	Avoid reuse, clear liquids at the detection area
	The muscle for detecting neurological effects is detached from the recording electrode	The surface electrodes of endotracheal tube should be located at the laryngeal anteromedian line
EMG signal is present while no nerve is detected	Consecutive “sequence” EMG response cannot be explained	Light anesthesia, with spontaneous activity of laryngeal muscle; the recording nerve or muscle is tracted by other nerve or muscle
	The detection current is too large	Direct detect the nerve trunk (1 mA is recommended) Adjust according to the anatomic structures and EMG signals during the surgery
	Artifacts occur in the non-neural traveling area	The surface electrode of endotracheal tube is placed too deeply
Under good V1 signal, there is the decrease in signal by >50% or LOS during the dissection of RLN	Anesthesia or muscle relaxation status changes	Avoid adding muscle relaxant
	Nerve transection injury	Check the nerve continuity
	Nerve injury not visible in naked eye	Locate the injury site and analyze the causes: traction, heat, suction, and/or thread-cutting injury
	Monitoring system failure	Recheck the electrode connections, the monitor, and interface-connector box
	Recording electrode displacement due to changes in head position or body position	Recheck the laryngoscope and adjust the endotracheal tube

EMG—electromyography; LOS—loss of signal; RLN—recurrent laryngeal nerve.

**Table 3:** General effects of drugs used in general anesthesia on ION modalities.

Drug Class	Example	Effects on IONM	Recommendation
Neuromuscular blocking agents	Rocuronium, Vecuronium	Suppresses EMG and MEP	Avoid during monitoring or use reversal agent
Inhalation Agents	Sevoflurane, Desflurane	Depresses evoked potentials	Use TIVA when MEP monitoring is critical
Opioids	Remifentanil, Sufentanil, Fentanyl	No interference with EMG or MEP	Preferred agent in IONM protocols
Benzodiazepines	Midazolam	Dose-dependent MEP suppression	Use cautiously; avoid bolus before stimulation
Intravenous Anesthetics	Propofol	Relatively stable EMG; high dose suppresses MEP	Preferred for TIVA; titrate to avoid deep suppression

## Abbreviations

The following abbreviations are used in this manuscript:

EMG	electromyography
IONM	intraoperative nerve monitoring
RLN	recurrent laryngeal nerve.
LOS	loss of signal

## References

[1] Pizzato M., Li M., Vignat J., Laversanne M., Singh D., La Vecchia C., Vaccarella S. (2022). The epidemiological landscape of thyroid cancer worldwide: GLOBOCAN estimates for incidence and mortality rates in 2020. Lancet Diabetes Endocrinol..

[2] Vaccarella S., Lortet-Tieulent J., Colombet M., Davies L., A Stiller C., Schüz J., Togawa K., Bray F., Franceschi S., Maso L.D. (2021). Global patterns and trends in incidence and mortality of thyroid cancer in children and adolescents: A population-based study. Lancet Diabetes Endocrinol..

[3] Kang M.J., Won Y.-J., Lee J.J., Jung K.-W., Kim H.-J., Kong H.-J., Im J.-S., Seo H.G. (2022). Cancer Statistics in Korea: Incidence, Mortality, Survival, and Prevalence in 2019. Cancer Res. Treat..

[4] Hassanipour S., Zare R., Shahedi A., Delam H. (2023). Survival rate of thyroid cancer in the Asian countries: A systematic review and meta-analysis study. Endocrine.

[5] Haugen B.R., Alexander E.K., Bible K.C., Doherty G.M., Mandel S.J., Nikiforov Y.E., Pacini F., Randolph G.W., Sawka A.M., Schlumberger M. (2016). 2015 American Thyroid Association Management Guidelines for Adult Patients with Thyroid Nodules and Differentiated Thyroid Cancer: The American Thyroid Association Guidelines Task Force on Thyroid Nodules and Differentiated Thyroid Cancer. Thyroid.

[6] Lukinović J., Bilić M. (2020). Overview of Thyroid Surgery Complications. Acta Clin. Croat..

[7] Christou N., Mathonnet M. (2013). Complications after total thyroidectomy. J. Visc. Surg..

[8] Sun H., Tian W., Jiang K., Chiang F., Wang P., Huang T., Zhu J., Qin J., Liu X. (2015). Clinical guidelines on intraoperative neuromonitoring during thyroid and parathyroid surgery. Ann. Transl. Med..

[9] Dralle H., Sekulla C., Haerting J., Timmermann W., Neumann H.J., Kruse E., Grond S., Mühlig H.P., Richter C., Voß J. (2004). Risk factors of paralysis and functional outcome after recurrent laryngeal nerve monitoring in thyroid surgery. Surgery.

[10] Gunn A., Oyekunle T., Stang M., Kazaure H., Scheri R. (2020). Recurrent Laryngeal Nerve Injury After Thyroid Surgery: An Analysis of 11,370 Patients. J. Surg. Res..

[11] Sanapala A., Nagaraju M., Rao L., Nalluri K. (2015). Management of bilateral recurrent laryngeal nerve paresis after thyroidectomy. Anesth. Essays Res..

[12] Snyder S.K., Lairmore T.C., Hendricks J.C., Roberts J.W. (2008). Elucidating mechanisms of recurrent laryngeal nerve injury during thyroidectomy and parathyroidectomy. J. Am. Coll. Surg..

[13] Barczyński M., Konturek A., Cichoń S. (2009). Randomized clinical trial of visualization versus neuromonitoring of recurrent laryngeal nerves during thyroidectomy. J. Br. Surg..

[14] Thomusch O., Sekulla C., Walls G., Machens A., Dralle H. (2002). Intraoperative neuromonitoring of surgery for benign goiter. Am. J. Surg..

[15] Dionigi G., Barczynski M., Chiang F.Y., Dralle H., Duran-Poveda M., Iacobone M., Lombardi C.P., Materazzi G., Mihai R., Randolph G.W. (2010). Why monitor the recurrent laryngeal nerve in thyroid surgery?. J. Endocrinol. Investig..

[16] Kazi R., Katna R., Dwivedi R.C. (2010). Minimal access thyroid surgery—A new dawn?. R. Coll. Surg. Engl..

[17] Dionigi G., Boni L., Rovera F., Bacuzzi A., Dionigi R. (2009). Neuromonitoring and video-assisted thyroidectomy: A prospective, randomized case-control evaluation. Surg. Endosc..

[18] Randolph G.W., Dralle H., Abdullah H., Barczynski M., Bellantone R., Brauckhoff M., Carnaille B., Cherenko S., Chiang F.-Y., Dionigi G. (2011). Electrophysiologic recurrent laryngeal nerve monitoring during thyroid and parathyroid surgery: International standards guideline statement. Laryngoscope.

[19] Sasaki C.T., Mitra S. (2001). Recurrent laryngeal nerve monitoring by cricopharyngeus contraction. Laryngoscope.

[20] Rea J.L., Khan A. (1998). Clinical evoked electromyography for recurrent laryngeal nerve preservation: Use of an endotracheal tube electrode and a postcricoid surface electrode. Laryngoscope.

[21] Brauckhoff M., Walls G., Brauckhoff K., Thanh P., Thomusch O., Dralle H. (2002). Identification of the non-recurrent inferior laryngeal nerve using intraoperative neurostimulation. Langenbecks Arch. Surg..

[22] Dackiw A.P., Rotstein L.E., Clark O.H. (2002). Computer-assisted evoked electromyography with stimulating surgical instruments for recurrent/external laryngeal nerve identification and preservation in thyroid and parathyroid operation. Surgery.

[23] Atlas G., Lee M. (2013). The neural integrity monitor electromyogram tracheal tube: Anesthetic considerations. J. Anaesthesiol. Clin. Pharmacol..

[24] Lu I., Chu K., Tsai C., Wu C., Kuo W., Chen H., Lee K., Chiang F. (2008). Optimal depth of NIM EMG endotracheal tube for intraoperative neuromonitoring of the recurrent laryngeal nerve during thyroidectomy. World J. Surg..

[25] You J.Y., Kim H.Y. (2021). Intraoperative Neuromonitoring during Thyroid Surgery. Int. J. Thyroidol..

[26] Chan W.F., Lo C.Y. (2006). Pitfalls of intraoperative neuromonitoring for predicting postoperative recurrent laryngeal nerve function during thyroidectomy. World J. Surg..

[27] Dhonneur G., Kirov K., Slavov V., Duvaldestin P. (1999). Effects of an intubating dose of succinylcholine and rocuronium on the larynx and diaphragm: An electromyographic study in humans. J. Am. Soc. Anesthesiol..

[28] Lundstrøm L.H., Møller A.M., Rosenstock C., Astrup G., Gätke M.R., Wetterslev J. (2009). Avoidance of neuromuscular blocking agents may increase the risk of difficult tracheal intubation: A cohort study of 103,812 consecutive adult patients recorded in the Danish Anaesthesia Database. Br. J. Anaesth..

[29] Bowman W. (2006). Neuromuscular block. Br. J. Pharmacol..

[30] Hunter J.M. (1996). Rocuronium: The newest aminosteroid neuromuscular blocking drug. Br. J. Anaesth..

[31] Gramstad L., Lilleaasen P. (1982). Dose-response relation for atracurium, Org NC45 and pancuronium. BJA Br. J. Anaesth..

[32] Mirakhur R.K. (1995). Dose-response and time-course of action of rocuronium bromide. Eur. J. Anaesthesiol. Suppl..

[33] Shahnawaz M.M., Shahjahan B., Sarwar S.S. (2011). Evaluation of intubating conditions after rocuronium bromide in adults induced with propofol or thiopentone sodium. J. Anaesthesiol. Clin. Pharmacol..

[34] Meistelman C., Plaud B., Donati F. (1992). Rocuronium (ORG 9426) neuromuscular blockade at the adductor muscles of the larynx and adductor pollicis in humans. Can. J. Anaesth..

[35] McCoy E.P., Mirakhur R.K., Maddineni V.R., Wierda J.M., Proost J.H. (1996). Pharmacokinetics of rocuronium after bolus and continuous infusion during halothane anaesthesia. Br. J. Anaesth..

[36] Wulf H., Ledowski T., Linstedt U., Proppe D., Sitzlack D. (1998). Neuromuscular blocking effects of rocuronium during desflurane, isoflurane, and sevoflurane anaesthesia. Can. J. Anaesth..

[37] Shanks C.A., Fragen R.J., Ling D. (1993). Continuous intravenous infusion of rocuronium (ORG 9426) in patients receiving balanced, enflurane, or isoflurane anesthesia. Anesthesiology.

[38] Oris B., Crul J.F., Vandermeersch E., Van Aken H., Van Egmond J., Sabbe M.B. (1993). Muscle paralysis by rocuronium during halothane, enflurane, isoflurane, and total intravenous anesthesia. Anesth. Analg..

[39] Kumar N., Mirakhur R.K., Symington M.J., McCarthy G.J. (1996). Potency and time course of action of rocuronium during desflurane and isoflurane anaesthesia. Br. J. Anaesth..

[40] Xue F.S., Liao X., Tong S.Y., Liu J.H., An G., Luo L.K. (1998). Dose-response and time-course of the effect of rocuronium bromide during sevoflurane anaesthesia. Anaesthesia.

[41] Li X., Zhang B., Yu L., Yang J., Tan H. (2020). Influence of Sevoflurane-Based Anesthesia versus Total Intravenous Anesthesia on Intraoperative Neuromonitoring during Thyroidectomy. Otolaryngol. Head Neck Surg..

[42] de Moraes N., Lanchote V., Filgueira G., Lopes B., Lepera J., Lauretti G. (2015). Impact of advanced age on the Pharmacokinetics and Pharmacodynamics of Rocuronium in patients undergoing elective surgery. Clin. Ther..

[43] Singh Y.N., Marshall I., Harvey A. (1979). Depression of transmitter release and postjunctional sensitivity during neuromuscular block produced by antibiotics. Br. J. Anaesth..

[44] Lee J.H., Lee S.I., Chung C.J., Lee J.H., Lee S.C., Choi S.R., Oh J.N., Bae J.Y. (2013). The synergistic effect of gentamicin and clindamycin on rocuronium-induced neuromuscular blockade. Korean J. Anesthesiol..

[45] Kim S.Y., Jin H.C., Lee J.S., Park J.H., Cho S.H., Kim S.I. (2000). Lidocaine and Verapamil Enhances Neuromuscular Block Induced by Rocuronium. Korean J. Anesthesiol..

[46] Durant N.N., Nguyen N., Katz R.L. (1984). Potentiation of neuromuscular blockade by verapamil. Anesthesiology.

[47] Bindra A., Bindu B., Rath G. (2017). Temperature management under general anesthesia: Compulsion or option. J. Anaesthesiol. Clin. Pharmacol..

[48] Heier T., Caldwell J.E., Warltier D.C. (2006). Impact of hypothermia on the response to neuromuscular blocking drugs. J. Am. Soc. Anesthesiol..

[49] Kovac A.L. (2009). Sugammadex: The first selective binding reversal agent for neuromuscular block. J. Clin. Anesth..

[50] Duvaldestin P., Kuizenga K., Saldien V., Claudius C., Servin F., Klein J., Debaene B., Heeringa M. (2010). A randomized, dose-response study of sugammadex given for the reversal of deep rocuronium- or vecuronium-induced neuromuscular blockade under sevoflurane anesthesia. Anesth. Analg..

[51] Pavoni V., Gianesello L., Martinelli C., Horton A., Nella A., Gori G., Simonelli M., De Scisciolo G. (2016). Recovery of laryngeal nerve function with sugammadex after rocuronium-induced profound neuromuscular block. J. Clin. Anesth..

[52] Gunes M.E., Dural A.C., Akarsu C., Guzey D., Sahbaz N.A., Tulubas E.K., Bulut S., Donmez T. (2019). Effect of intraoperative neuromonitoring on efficacy and safety using sugammadex in thyroid surgery: Randomized clinical trial. Ann. Surg. Treat. Res..

[53] Chai Y.J., Lee J., Won D., Lee J., Hwang J., Kim T.K., Chang J., Kim H., Yang H.J., Min S. (2021). Comparison of Sugammadex Dose for Intraoperative Neuromonitoring in Thyroid Surgery: A Randomized Controlled Trial. Laryngoscope.

[54] Lu I.-C., Hsu C.-D., Chang P.-Y., Wu S.-H., Huang T.-Y., Lin Y.-C., Ko H.-Y., Dionigi G., Chai Y.J., Chiang F.-Y. (2022). A Surgeon-Centered Neuromuscular Block Protocol Improving Intraoperative Neuromonitoring Outcome of Thyroid Surgery. Front. Endocrinol..

[55] Dionigi G., Bacuzzi A., Boni L., Rovera F., Dionigi R. (2008). What is the learning curve for intraoperative neuromonitoring in thyroid surgery?. Int. J. Surg..

[56] Apfelbaum J.L., Hagberg C.A., Connis R.T., Abdelmalak B.B., Agarkar M., Dutton R.P., Fiadjoe J.E., Greif R., Klock P.A., Mercier D. (2022). 2022 American Society of Anesthesiologists Practice Guidelines for Management of the Difficult Airway. Anesthesiology.

[57] Bouaggad A., Nejmi S.E., Bouderka M.A., Abbassi O. (2004). Prediction of difficult tracheal intubation in thyroid surgery. Anesth. Analg..

[58] Liu D.-X., Ye Y., Zhu Y.-H., Li J., He H.-Y., Dong L., Zhu Z.-Q. (2019). Intubation of non-difficult airways using video laryngoscope versus direct laryngoscope: A randomized, parallel-group study. BMC Anesthesiol..

[59] Kriege M., Hilt J.A., Dette F., Wittenmeier E., Meuser R., Staubitz J.I., Musholt T.J. (2023). Impact of direct laryngoscopy vs. videolaryngoscopy on signal quality of recurrent laryngeal nerve monitoring in thyroid surgery: A randomised parallel group trial. Anaesthesia.

[60] Conrardy P.A., Goodman L.R., Lainge F., Singer M.M. (1976). Alteration of endotracheal tube position flexion and extension of the neck. Crit. Care Med..

[61] Yap S.J., Morris R.W., Pybus D.A. (1994). Alterations in endotracheal tube position during general anaesthesia. Anaesth. Intensive Care.

[62] Won D., Lee J.-M., Lee J., Chai Y.J., Hwang J.-Y., Kim T.K., Chang J.-E., Kim H., Kim M.J., Min S.-W. (2024). Usefulness of video laryngoscopy in tracheal intubation at thyroid surgical position for intraoperative neuromonitoring. Sci. Rep..

[63] Schneider R., Sekulla C., Machens A., Lorenz K., Thanh P.N., Dralle H. (2016). Dynamics of loss and recovery of the nerve monitoring signal during thyroidectomy predict early postoperative vocal fold function. Head Neck.

[64] Shin S.-C., Lee B.-J. (2020). A New Era of Intraoperative Neuromonitoring: Beyond the Electromyography Endotracheal Tube During Thyroid Surgery. Clin. Exp. Otorhinolaryngol..

[65] Dralle H., Sekulla C., Lorenz K., Brauckhoff M., Machens A. (2008). German IONM Study Group Intraoperative monitoring of the recurrent laryngeal nerve in thyroid surgery. World J. Surg..

[66] Lee H.S., Oh J., Kim S.W., Jeong Y.W., Wu C., Chiang F., Lee K.D. (2020). Intraoperative neuromonitoring of recurrent laryngeal nerve during thyroidectomy with adhesive skin electrodes. World J. Surg..

[67] Chiang F., Lu I., Chang P., Dionigi G., Randolph G.W., Sun H., Lee K., Tae K., Ji Y.B., Kim S.W. (2017). Comparison of EMG signals recorded by surface electrodes on endotracheal tube and thyroid cartilage during monitored thyroidectomy. Kaohsiung J. Med. Sci..

[68] Schneider R., Randolph G.W., Sekulla C., Phelan E., Thanh P.N., Bucher M., Machens A., Dralle H., Lorenz K. (2013). Continuous intraoperative vagus nerve stimulation for identification of imminent recurrent laryngeal nerve injury. Head Neck.

[69] Randolph G.W., Kamani D., Wu C.-W., Schneider R. (2021). Surgical anatomy and monitoring of the recurrent laryngeal nerve. Surgery of the Thyroid and Parathyroid Glands.

